# Follow-Up Survey for Conservatively Managed Ureteric Stones

**DOI:** 10.7759/cureus.66845

**Published:** 2024-08-14

**Authors:** Farhan Jarral, Abdelrahman Hamdy, Jakub Wrazen, Guleed Mohamed, Osama Abusanad

**Affiliations:** 1 Urology, Doncaster Royal Infirmary, Doncaster, GBR; 2 General Surgery, Doncaster Royal Infirmary, Doncaster, GBR; 3 Urology, Mid-Yorkshire NHS Trust, Leeds, GBR; 4 Urology, Chesterfield Royal Hospital, Chesterfield, GBR

**Keywords:** endourology, conservative stone management, distal ureteric stones, lowers ureteric stones, ureteric stones

## Abstract

Introduction

Currently, there are no agreed-upon investigations and follow-up guidelines for the conservative management of ureteric stones. This study used common themes identified in previous works to investigate whether there is a consensus amongst urology consultants in the United Kingdom.

Methods

This was a questionnaire-based survey study. An online questionnaire was disseminated nationally to urological consultants practicing in the United Kingdom to explore a range of common factors. The initial sample size was 81 UK-based urological consultants with an interest in endourology and stone surgery. Of the initial 81, 20 participants did not complete the survey and therefore the final sample size was 61. Descriptive analysis was used to analyze the data.

Results

Our survey found that the main factors influencing the follow-up of conservatively managed ureteric stones were stone size 98% (60), stone location 92% (56), and the degree of altered renal function 79% (48). Regardless of stone size, most participants chose to follow up at 2-4 weeks with asymptomatic patients requiring imaging with discrepancies about the modality. Regarding biochemical markers, most participants only repeated renal function tests if this was deranged on presentation. Calcium and uric acid levels were checked regularly. Diclofenac was the analgesia of choice 93% (55). Regarding the availability of acute ESWL services, over half (59%) were able to offer acute ESWL within the week. The majority offer services at least three or more lists per week.

Conclusion

Our results demonstrate that there is still no overarching consensus in the follow-up of conservatively managed ureteric stones. Several factors backed by high-level evidence are already consistent across the population of urology consultants and considered "best practice." However, before any all-encompassing national guidelines are formalized, further studies in the form of randomized control trials will be needed to yield high-level evidence.

## Introduction

Renal and ureteric stones (urolithiasis) pose a large economic and clinical burden to the healthcare system of the United Kingdom. An increasing trend in the incidence and prevalence of renal and ureteric stones. The National Institute of Clinical Excellence (NICE) reports a 70% increase over a 15-year period (2000-2015) in the number of hospital episodes from 51,035 episodes to 86,742 episodes [1].

Conservative management is possible in patients with small, distal ureteric stones. A UK-based study demonstrated a spontaneous passage rate of 89% in patients with small, distal ureteric stones [2]. Certain factors such as stone size [3] position in the ureter [4], or degree of symptoms [5] are well established and important in initial decision-making. Meanwhile, other decisions such as time to follow up or interval imaging are less universally agreed upon. The lack of structured guidelines on ureteric stone follow-up may be a result of insufficient evidence or inconsistencies in service provision. Collating common practices among urology consultants in the UK may shed light on areas of similarities and differences with the aim of establishing a consensus in practice. The lack of structured guidance on ureteric stone follow-up has created a gap in the literature. Equally, there may already be consensus in certain areas of managing this cohort of patients, closing the gap in the literature, and providing a level of evidence that could form part of any future structured guidelines. Establishing clear guidelines can reduce the need for specialist input by allowing simple cases to be triaged and managed by the emergency services. Finally, and most importantly, unifying all the best available evidence into one clear guideline should improve patient safety and outcomes.

## Materials and methods

This was an online questionnaire-based survey study. A literature search demonstrated a lack of surveys regarding the follow-up of conservatively managed ureteric stones. Using principles mentioned in the CHERRIES checklist, a 10-item survey was designed; this was analyzed by consultant urologists with a specialist interest in stone surgery and was piloted locally prior to being sent to all voluntarily participating urological consultants based in the United Kingdom. Participants were allocated four weeks to respond, with reminders being sent every two weeks.

The sample size was 81 UK-based consultant urological surgeons. The inclusion criteria were completed questionnaires from consultant urological surgeons with a specialist interest in endourology and stones and currently practicing in the National Health Service (NHS) across the whole of the United Kingdom. 

Of the 81 participants to whom the questionnaire was sent, we received completed responses from 75% (61). Therefore the final sample size was 61 which is represented as 100% in the dataset. Descriptive analysis was used in our study as the percentage of participants opting for their respective choices. Moreover, data visualization was used in the form of tables demonstrating the responses.

The questions were designed by one of the authors around previously established factors such as stones sized below and above 5mm (Table 1). These were then trialed locally with colleagues before the survey was then disseminated to consultants across the United Kingdom. Participants had the option of selecting multiple choices under a single question to allow for flexibility and give a more accurate reflection. The option of stating any further responses deemed relevant by the respondents under the heading "other" was offered on each question. The following questions were asked.

**Table 1:** Questions asked

Questions asked
1. What are the factors that influence your follow-up plan for patients with ureteric stone, opted for conservative management?
2. When do you follow up symptomatic patients with obstructing (5mm or less) ureteric stone, opted for conservative management?
3. When do you follow up symptomatic patients with obstructing (>5mm) ureteric stone, opted for conservative management?
4. During follow-up of the (5mm or less) ureteric stone, would you image an asymptomatic patient?
5. During follow-up of the (>5mm) ureteric stone, would you image an asymptomatic patient?
6. During follow-up, do you repeat renal function bloods to evaluate renal function changes?
7. During follow-up, do you check for calcium and uric acid?
8. What are the routine biochemical evaluations for new patients with ureteric stones in your hospital?
9. What is the first choice of analgesia for ureteric colic in your hospital?
10. Do you have the facility for acute SWL service for treating obstructing ureteric stones?

## Results

The first question was designed to assess consensus opinion across broad, previously identified factors in the conservative approach to ureteric stones [5]. The survey demonstrates that the three most prevalent factors influencing the follow-up of patients with conservatively managed ureteric stones were: stone size 98% (60), location of the stone 92% (56), and degree of altered renal function at presentation 79% (48) (Table 2).

**Table 2:** Question 1 - What are the factors that influence your follow-up plan for patients with ureteric stone, opted for conservative management? The percentages indicated demonstrate the number of participants opting for the respective choices

	Responses (61)	Percentage
Size of stone	60	98%
Location of the stone	56	92%
Outpatient department capacity	3	5%
Severity of symptoms	43	70%
Degree of hydronephrosis	31	51%
Presence of renal stones	11	18%
Degree of altered renal function at presentation	48	79%

The second (Table 3) and third (Table 4) questions assessed the most frequently used follow-up intervals for symptomatic stones of different sizes. Around half of the consultants choose to follow up patients at 2-4 weeks regardless of stone size, 51% (31) for ≤5mm and 56% (34) for >5mm, respectively. This is roughly in line with the current British Association of Urological Surgeons (BAUS) recommendations of active treatment if the stone has not passed in 2-3 weeks [6].

**Table 3:** Question 2 - When do you follow up symptomatic patients with obstructing (5mm or less) ureteric stone, opted for conservative management? The percentages indicated demonstrate the number of participants opting for the respective choices

	Responses (61)	Percentage
Within 2 weeks	3	5%
2-4 weeks	31	51%
4-6 weeks	23	38%
Other	4	4%

**Table 4:** Question 3 - When do you follow up symptomatic patients with obstructing (>5mm) ureteric stone, opted for conservative management? The percentages indicated demonstrate the number of participants opting for the respective choices

	Responses (61)	Percentage
Within 2 weeks	11	18%
2-4 weeks	34	56%
4-6 weeks	10	16%
Other	6	10%

Questions four and five looked at the choice of imaging modality in asymptomatic patients during follow-up based on the size of the stone ≤5mm or >5mm, respectively. This was understandably more nuanced with each imaging modality having its own benefits and downsides. The most widely selected imaging modality when following up asymptomatic patients with stones ≤5mm was X-ray KUB in radio-opaque stones 36% (22), with the second most common modality being non-contrast CT KUB 28% (17). About 11% (7) responded that they would choose KUB USS to assess for hydronephrosis and 5% (3) chose no imaging modality (Table 5).

**Table 5:** Question 4 - During follow-up of the (5mm or less) ureteric stone, would you image an asymptomatic patient? The percentages indicated demonstrate the number of participants opting for the respective choices

	Responses (61)	Percentage
Yes, X-ray KUB (in radio-opaque stone)	22	36%
Yes, KUB USS (assess hydronephrosis)	7	11%
Yes, Non-contrast CT KUB	17	28%
No	3	5%
Other	12	20%

Their responses were similar for stones >5mm. The majority of respondents selected X-ray KUB 43% (26), with the second most common imaging modality being non-contract CT KUB again 33% (20). Only 8% (5) and 2% (1) said they would use KUB USS and not image, respectively (Table 6).

**Table 6:** Question 5 - During follow-up of the (>5mm) ureteric stone, would you image an asymptomatic patient? The percentages indicated demonstrate the number of participants opting for the respective choices

	Responses (61)	Percentage
Yes, X-ray KUB (in radio-opaque stone)	26	43%
Yes, KUB USS (assess hydronephrosis)	5	8%
Yes, Non-contrast CT KUB	20	33%
No	1	2%
Other	9	15%

The following three questions assessed approaches to investigating common biochemical markers. A total of 49 consultants reported they repeat renal function at follow-up. Of these, only 5% (3) repeat renal functions routinely and 75% (46) only if altered at initial presentation. About 18% (11) of consultants do not repeat renal function blood tests (Table 7).

**Table 7:** Question 6 - During follow-up, do you repeat renal function bloods to evaluate renal function changes? The percentages indicated demonstrate the number of participants opting for the respective choices

	Responses (61)	Percentage
Yes, routinely	3	5%
Yes, only if altered at initial presentation	46	75%
No	11	18%
Other	1	2%

With regards to measuring serum calcium and uric acid during follow-up, question 7 demonstrated that 75% (46) checked both and 16% (10) only measured serum calcium. Only 5% (3) consultants said they did not check blood results (Table 8). The remaining respondents checked serum calcium and uric acid in addition to parathyroid hormone (PTH), vitamin D, and serum glucose.

**Table 8:** Question 7 - During follow-up, do you check for calcium and uric acid? The percentages indicated demonstrate the number of participants opting for the respective choices

	Responses (61)	Percentage
Yes, both calcium and uric acid	46	75%
Yes, calcium only	10	16%
No	3	5%
Other	2	3%

Conversely, question 8 assessed the routine biochemical workup of the patient’s urinary tract stones at first presentation (Table 9). Of the respondents, 100% (61), 82% (50), and 38% (23) checked for serum calcium, uric acid, and urinary pH, respectively. Of the other responses, 13% (8) included: 24-hour urine in <25 years old patients, vitamin D and PTH, triglyceride, high-density lipoprotein (HDL), and random glucose.

**Table 9:** Question 8 - What are the routine biochemical evaluations for new patients with ureteric stones in your hospital? The percentages indicated demonstrate the number of participants opting for the respective choices

	Responses (61)	Percentage
Serum calcium	61	100%
Serum uric acid	50	82%
Urine pH	23	38%
Urine spot test	0	0%
Other	8	13%

Ensuring adequate analgesia is provided may prove to be difficult as all patients experience pain differently. National Institute for Health and Care Excellence (NICE) guidelines [7] recommend that any patient aged 16 years onwards with suspected renal or ureteric colic be offered nonsteroidal anti-inflammatory drugs by any route unless contraindicated or are not given sufficient pain relief. Responses to question 9 revealed the first choice of analgesia for ureteric colic was diclofenac at 93% (57) followed by ibuprofen at 3% (2), paracetamol and ketorolac at 2% (1) each (Table 10).

**Table 10:** Question 9 - What is the first choice of analgesia for ureteric colic in your hospital? The percentages indicated demonstrate the number of participants opting for the respective choices

	Responses (61)	Percentage
Paracetamol	1	2%
Ibuprofen	2	3%
Diclofenac	55	93%
Codeine	0	0%
Morphine	0	0%
Other	3	5%

Finally, the provision of extracorporeal shock-wave lithotripsy (ESWL) was assessed in question 10. As per NICE, ESWL is a recognized option for treating patients with obstructing ureteric stones [8]. Unfortunately, according to the survey, 30% (18) of respondents said they do not have this service in the acute setting. Of those that responded with yes, there was a difference in the availability of this service at the respondent’s center. Once a week 8% (5), twice a week 7% (4), and three or more shock-wave lithotripsy (SWL) lists per week 44% (27) (Table 11).

**Table 11:** Question 10 - Do you have the facility for acute ESWL service for treating obstructing ureteric stones? The percentages indicated demonstrate the number of participants opting for the respective choices ESWL: extracorporeal shock-wave lithotripsy

	Responses (61)	Percentage
No	18	30%
Yes, once a week SWL list	5	8%
Yes, twice a week	4	7%
Yes, three or more SWL lists per week	27	44%
Other	7	11%

## Discussion

Although this survey demonstrated a certain variability in UK-based practice amongst urological consultants, there was consensus on several factors. An obvious limitation of this study is the difficulty of weighing expert consensus as it is based on subjective experience and anecdotal in nature [9]. Previous works have proposed different thresholds for considering a consensus to be significant with the most frequent proportion being around 70-80% [10]. Going off this threshold, certain factors and practices are well established. About 98% (60) of consultants reported that stone size and 92% (56) cited position affected their decision-making. These were backed by well-established high-quality evidence and are essentially seen as best practices despite not being formalized. Other factors that fell into this category included using diclofenac for pain relief and investigating calcium + uric acid. The third most frequently highlighted factor was the degree of renal dysfunction in the first presentation. While this is clearly a useful metric, quantifying a cut-off point for guidelines may be difficult as the change in outcomes likely varies linearly with the degree of dysfunction. Future studies could look at renal function thresholds at which patient outcomes would be unchanged at the time of established follow-up. Otherwise, if these guidelines are to primarily guide non-specialist physicians any degree of dysfunction may have to be viewed as significant. On the other hand, factors like co-existing renal stones or outpatient capacity were generally considered unimportant and as such would not be first-line targets for further investigation.

Other factors were less consistent across our cohort. While most respondents agreed that patients need follow-up imaging regardless of stone size (75% (46) and 84% (51) for <5mm and >5mm, respectively); there was no strong consensus about imaging modality. All three named imaging modalities have their advantages and are evidence-based [11,12]. The lack of consensus may be due to availability but given that each modality has its clear uses and can be used appropriately this could be left to the discretion of the requesting clinician. Furthermore, there was no consensus on the time to follow up with around half of the respondents agreeing with 2-4 weeks. As mentioned earlier BAUS currently recommends active treatment if the stone has not passed in 2-3 weeks [6]. However, this seems to be based on the average chance of stones passing within a certain time frame rather than the actual examination of patient outcomes for different follow-up times and could be a target for further investigation. As a first step, this would be relatively easy to assess retrospectively and if significant differences are found it could be worth investigating further.

Finally, do we even need a consensus [13]? Even with well-established guidelines based on high-level evidence, there is always room for nuance and in reality, practice differs between individual physicians based on a multitude of factors such as personal preferences or availability of different services. This was clearly reflected in the responses to question 10 where a third of surveyed consultants did not have access to the treatment of choice in the acute setting. As a result, many trusts already have local guidelines on managing ureteric stones. If patient outcomes are good and the nuances of conservative management of ureteric stones are already handled by respective departments, trying to standardize management may not only be difficult but also unnecessary.

However one may argue that to deliver the same standard of high-level care to all patients, a formalized set of guidelines should be established. An initial study to establish consensus amongst urological consultants could highlight good practices from one region or center and allow them to be enacted throughout the UK.

As with all research, this study also has multiple limitations. First, the final sample size was 61 this is a relatively small sample size which therefore may not be representative of all urological consultants with an interest in stone surgery. Second, as the study is based on a non-validated questionnaire; we are not able to probe the data and analyze the rationale of the respondents. Therefore, responses may not be evidence-based but rather due to personal professional experience and/or anecdotal.

## Conclusions

Though there is some agreement amongst urologists regarding certain factors, there is no unanimous consensus on the follow-up of conservatively managed ureteric stones.

Broadly speaking, factors including analgesia of choice and laboratory investigations were largely agreed upon. However, approaches were less consistent in the choice of follow-up imaging modality, ESWL use, and time frame for follow-up. Rather than focusing on largely agreed-upon areas, we suggest further studies look into the areas of discrepancy. This would enable us to form a more holistic approach to drawing up a consensus in the follow-up of patients with ureteric stones being managed conservatively. Further work using the Delphi methodology would be useful to validate these findings. If consistent, these could then be discussed with bodies such as the BAUS for more robust targeted studies with the ultimate aim of developing consensus and structured guidelines.

## Appendices

**Table 12:** Follow-up survey for conservatively managed ureteric stones

Follow-up survey for conservatively managed ureteric stones
1. What are the factors that influence your follow up plan for patients with ureteric stone, opted for conservative management?
A) Size of stone
B) Location of the stone
C) Outpatient department capacity
D) Severity of symptoms
E) Degree of hydronephrosis
F) Presence of renal stones
G) Degree of altered renal function at presentation
2. When do you follow up symptomatic patient with obstructing (5mm or less) ureteric stone, opted for conservative management?
A) Within 2 weeks
B) 2-4 weeks
C) 4-6 weeks
D) Other
3. When do you follow up symptomatic patient with obstructing (>5mm) ureteric stone, opted for conservative management?
A) Within 2 weeks
B) 2-4 weeks
C) 4-6 weeks
D) Other
4. During follow up of the (5mm or less) ureteric stone, would you image an asymptomatic patient?
A) Yes, X-ray KUB (in radio opaque stone)
B) Yes, KUB USS (assess hydronephrosis)
C) Yes, non-contrast CT KUB
D) No
E) Other
5. During follow up of the (>5mm) ureteric stone, would you image an asymptomatic patient?
A) Yes, X-ray KUB (in radio-opaque stone)
B) Yes, KUB USS (assess hydronephrosis)
C) Yes, Non-contrast CT KUB
D) No
E) Other
6. During follow-up, do you repeat renal function bloods to evaluate renal function changes?
A) Yes, routinely
B) Yes, only if altered at initial presentation
C) No
D) Other
7. During follow up, do you check for calcium and uric acid?
A) Yes, both calcium and uric acid
B) Yes, calcium only
C) No
D) Other
8. What are the routine biochemical evaluations for new patients with urinary in your hospital?
A) Serum calcium
B) Serum uric acid
C) Urine pH
D) Urine spot test
E) 24-hour urine collection
9. What is the first choice of analgesia for ureteric colic in your hospital?
A) Paracetamol
B) Ibuprofen
C) Diclofenac
D) Codeine
E) Morphine
F) Other
10. Do you have the facility for Acute SWL service for treating obstructing ureteric stone?
A) No
B) Yes, once a week SWL list
C) Yes, twice a week
D) Yes, 3 or more SWL lists per week
E) Other
